# Remote preparation for single-photon two-qubit hybrid state with hyperentanglement via linear-optical elements

**DOI:** 10.1038/s41598-018-37159-5

**Published:** 2019-03-20

**Authors:** Xian-Fang Jiao, Ping Zhou, Shu-Xin Lv, Zhi-Yong Wang

**Affiliations:** 1 College of Science, Guangxi University for Nationalities, Nanning, 530006 People’s Republic of China; 2 Key lab of quantum information and quantum optics, Guangxi University for Nationalities, Nanning, 530006 People’s Republic of China; 3 Guangxi Key Laboratory of Hybrid Computational and IC Design Analysis, Nanning, 530006 People’s Republic of China

## Abstract

Linear-optical-based quantum information processing has attached much attention since photon is an ideal medium for transmitting quantum information remotely. Until now, there are some important works in quantum state remote preparation, the method for reconstructing quantum state deterministically via linear optics. However, most of the methods are protocols to prepare single-qubit states remotely via linear-optical elements. In this article, we investigate the methods to prepare two-qubit hybrid states remotely. We present a deterministic remote state preparation scheme for an arbitrary two-qubit hybrid state via a hyperentangled Bell state, resorting to linear-optical elements only. The sender rotates the spatial-mode state and polarization state of the hyperentangled photon respectively in accordance with his knowledge of the two-qubit hybrid state, and the receiver can reconstruct the original two-qubit hybrid state by applying appropriate recovery operations. Moreover, we discuss the remote state preparation scheme for the two-qubit hybrid state via partially hyperentangled Bell state.

## Introduction

Quantum entanglement is a precious resource in quantum communication^1–8^. The utilization of quantum states and quantum entangled states in quantum communication allows some new methods for quantum information transmitting^4–21^. Quantum communication can utilize the nonlocality of previously shared quantum entangled states to prepare an arbitrary unknown or known state on remote receiver’s quantum system without physically sending the original quantum system^22–26^. In quantum teleportation the agent can teleport an unknown single-qubit state via one ebit of quantum entanglement(a maximally entangled state of two qubits) and two bits of classical communication^4,5^. Remote state preparation may be understood as teleport the known state. In remote state preparation, the sender is supposed to have the complete information of the state to be transmitted^6–8^. “In 2000, Bennett, Pati and Lo showed that the quantum entanglement and classical communication cost can be reduced since the sender can perform the proper measurement on his entangled particle in accordance with his information of original state. Moreover, they studied the trade-off in remote state preparation between the required entanglement and the classical communication. Remote state preparation has been attached much interest since its important application in distributed quantum communication and large-scale quantum communication network^27–33^.

Recently, hyperentangled states which entangle in multiple degrees of freedom have been studied by some groups^34–50^. Quantum communication and quantum computation via hyperentangled state which processes quantum information simultaneously in multiple degrees of freedom can improve the channel capacity of long distance quantum communication and speedup quantum computation^51–57^. Kwiat showed that the application of multiply-entangled photons can assist us to implement Bell-state analysis^51^. In 2007, Wei *et al*. investigate hyperentangled Bell-state analysis and propose a protocol to group 16 hyperentangled Bell-state into 7 classes^52^. In 2010, Sheng *et al*. presented a protocol for complete hyperentangle Bell-state analysis which can distinguish the hyperentangled Bell states completely^53^. Moreover, they present protocols for quantum teleportation and entanglement swapping with hyperentangled states. Wang *et al*. studied quantum repeater which can convert the spatial entanglement into the polarization entanglement in 2012^54^. In 2013, Ren *et al*. proposed protocol for hyperentanglement concentration of two-photon four-qubit systems via parameter-splitting method^55^. Ren *et al*. introduced the concept of hyperparallel quantum computation which can perform universal quantum operation in multiple degrees of freedom (DOFs)^56^. In 2015, Ren, Wang and Deng presented two universal hyperparllel hybrid quantum gates on photon systems in multiple DOFs^57^. In 2015, quantum teleportation of multiple DOFs of a single photon has been experimentally realized via hyperentangled states^58^. Up to now, 18-qubit Greenberger- Horne- Zeilinger entanglement has been experimentally demonstrated via three degrees of freedom of six photons^59^.

In the implementation of quantum state remote preparation, transmitting quantum state remotely via linear-optical elements has attached a great deal of interest since photon is an ideal information carrier for long-distance quantum communication^60–66^. In 2010, Sheng and Deng proposed the protocol for deterministic entanglement purification with linear-optical elements only which is different from the previous schemes since the entanglement purification protocol works in a deterministic way^60^. In 2014, Li *et al*. presented an efficient protocol to concentrate partially entangled *chi*-type states with linear-optical elements^61^. In 2007, Liu *et al*. demonstrated an experiment to prepare single-photon polarization state remotely via linear-optical elements^62^. In 2010, Wu *et al*. presented a deterministic remote preparation scheme for arbitrary single-qubit pure or mixed state with linear optics^63^. Barreiro *et al*. reported the preparation of hybrid entangled state $\frac{1}{\sqrt{2}}(|Hl\rangle \pm |Vr\rangle )$ via the hyperentangled state $\frac{(|HH\rangle +|VV\rangle )}{\sqrt{2}}\otimes \frac{(|lr\rangle +|rl\rangle )}{\sqrt{2}}$, where |*H*〉 (|*V*〉) denotes the horizontal (vertical) polarization of photon and |*l*〉 (|*r*〉) refers to the paraxial spatial mode carrying +$\hslash$(−$\hslash$) units of orbital angular momentum. Moreover they discuss the protocol to prepare two-qubit hybrid state remotely via positive operator-valued measure with 4 cbits and 2 ebits^64^.

Different to previously remote state preparation in which single-qubit states are remotely prepared via linear-optical elements, parallel remote state preparation prepares quantum states which are encoded in multiple DOFs^62,63,65^. In this work, we present a protocol to prepare arbitrary two-qubit states remotely via hyperentangled states resorting only to linear-optical elements. The sender need only to rotate the polarization and spatial-mode state of single photon in accordance with the information of the original two-qubit state, and the two-qubit state can be remotely prepared at the receiver’s quantum system with 4 cbits and 2 ebits. Moreover, we discuss the remote state preparation protocol for two-qubit hybrid states via linear-optical elements with partially hyperentangled states.

## Results

### Two-qubit hybrid state remote preparation with a hyperentangled Bell state

To present the principle of two-qubit hybrid state remote preparation clearly, we first present the protocol for remote preparation of the single-photon two-qubit hybrid state via a hyperentangled Bell state, and then generalize it to the case with a partially hyperentangled Bell state.

An arbitrary single-photon two-qubit hybrid state can be described as^64^

1 $$|{\rm{\Psi }}\rangle ={\alpha }_{00}|H{a}_{0}\rangle +{\alpha }_{01}|H{a}_{1}\rangle +{\alpha }_{10}|V{a}_{0}\rangle +{\alpha }_{11}|V{a}_{1}\rangle $$

where |*H*〉, |*V*〉 represent horizontal polarizations and vertical polarizations of photons. |*a*_0_〉, |*a*_1_〉 represent two spatial modes of photon A. *α*_00_, *α*_01_, *α*_10_, *α*_11_ are arbitrary complex numbers which satisfy the normalization condition |*α*_00_|^2^ + |*α*_01_|^2^ + |*α*_10_|^2^ + |*α*_11_|^2^ = 1. Similar to ref.^6^, the coefficients *α*_00_, *α*_01_, *α*_10_, *α*_11_ are completely known by the sender Alice but unknown by the receiver Bob.

To prepare the two-qubit hybrid state remotely, the sender (Alice) and the receiver (Bob) share a two-photon four-qubit hyperentangled Bell state^47^

2 $$\begin{array}{rcl}|{\rm{\Phi }}{\rangle }_{AB} & = & \frac{1}{2}(|HH\rangle +|VV\rangle )\otimes (|{a}_{0}{b}_{0}\rangle +|{a}_{1}{b}_{1}\rangle )\\  & = & \frac{1}{2}(|H{a}_{0}\rangle |H{b}_{0}\rangle +|H{a}_{1}\rangle |H{b}_{1}\rangle \\  &  & +\,|V{a}_{0}\rangle |V{b}_{0}\rangle +|V{a}_{1}\rangle |V{b}_{1}\rangle ).\end{array}$$

Here photon A belongs to Alice and photon B belongs to Bob. |*a*_0_〉, |*a*_1_〉 are two spatial modes of photon A and |*b*_0_〉, |*b*_1_〉 are two spatial modes of photon B.

To prepare the two-qubit state remotely, Alice pre-adjust the original quantum channel to a target channel by rotation the polarization and spatial-mode state of photon A in accordance with his knowledge of the two-qubit state |Ψ〉, and then perform single-qubit measurement on his particle A. The two-qubit hybrid state |Ψ〉 can be remotely prepared onto the receiver’s hyperentangled photon B if the receiver cooperates with the sender.

**Figure 1:**
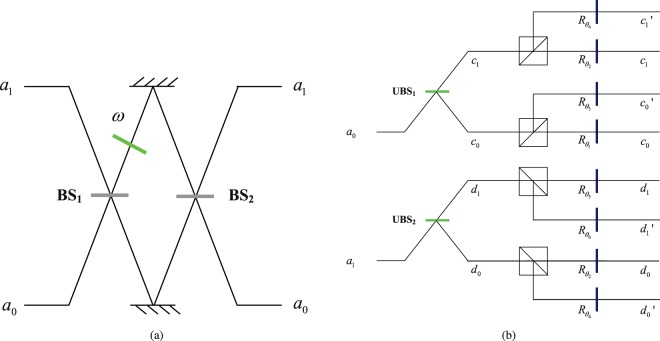
(**a**) Schematic diagram for manipulating the spatial-mode state of photon via unbalanced beam splitter (UBS). *ω* is a wave plate which adds a wave shift between the two spatial modes. (**b**) Quantum circuit for manipulation the spatial-mode and polarization states of photon A via linear-optical elements. *a*_0_, *a*_1_ are two spatial modes of photon A. *UBS*_1_, *UBS*_2_ represent two unbalanced beam splitters with the reflect coefficient ${R}_{1}=\sqrt{|{\alpha }_{00}{|}^{2}+|{\alpha }_{10}{|}^{2}}$, ${R}_{2}=\sqrt{|{\alpha }_{01}{|}^{2}+|{\alpha }_{11}{|}^{2}}$. The wave plate ${R}_{{\theta }_{l}}$ $(l=1,2,\ldots ,6)$ rotates the photon horizontal and vertical polarizations with angle *θ*_*l*_.

The setup for two-qubit hybrid state |Ψ〉 remote preparation with hyperentangled Bell state |Φ〉 is shown in Fig. 1. To pre-adjust the hyperentangled state to the target quantum channel, the spatial-mode state and polarization state of hyperentangled photon is rotated according to the sender’s information of prepared state following some ideas in quantum state initialization^67^. Alice rotates the spatial-mode state of photon A on spatial modes *a*_0_, *a*_1_ via unbalance beam splitters *UBS*_1_, *UBS*_2_ with reflect coefficient ${R}_{1}=\sqrt{|{\alpha }_{00}{|}^{2}+|{\alpha }_{10}{|}^{2}}$, ${R}_{2}=\sqrt{|{\alpha }_{01}{|}^{2}+|{\alpha }_{11}{|}^{2}}$^64^. The state of composite system composed of photons A, B is transformed from |Φ〉_*AB*_ to |Φ_1_〉_*AB*_ after photon A passes through *UBS*_1_, *UBS*_2_. (neglect a whole factor $\tfrac{1}{2}$)

3 $$\begin{array}{rcl}|{{\rm{\Phi }}}_{1}{\rangle }_{AB} & = & (\sqrt{{\alpha }_{00}^{2}+{\alpha }_{10}^{2}}|{c}_{0}\rangle +\sqrt{{\alpha }_{01}^{2}+{\alpha }_{11}^{2}}|{c}_{1}\rangle )|H\rangle |H{b}_{0}\rangle \\  &  & +\,(\sqrt{{\alpha }_{01}^{2}+{\alpha }_{11}^{2}}|{d}_{0}\rangle +\sqrt{{\alpha }_{00}^{2}+{\alpha }_{10}^{2}}|{d}_{1}\rangle )|H\rangle |H{b}_{1}\rangle \\  &  & +\,(\sqrt{{\alpha }_{00}^{2}+{\alpha }_{10}^{2}}|{c}_{0}\rangle +\sqrt{{\alpha }_{01}^{2}+{\alpha }_{11}^{2}}|{c}_{1}\rangle )|V\rangle |V{b}_{0}\rangle \\  &  & +\,(\sqrt{{\alpha }_{01}^{2}+{\alpha }_{11}^{2}}|{d}_{0}\rangle +\sqrt{{\alpha }_{00}^{2}+{\alpha }_{10}^{2}}|{d}_{1}\rangle )|V\rangle |V{b}_{1}\rangle .\end{array}$$

To pre-adjust the hyperentangled state to the target quantum channel, Alice rotates the polarization state of photon A in spatial modes ${c}_{0},{c^{\prime} }_{0}$, ${d}_{0},{d^{\prime} }_{0}$, ${c}_{1},{c^{\prime} }_{1}$, ${d}_{1},{d^{\prime} }_{1}$ via wave plates *R*(*θ*_*l*_) $(l=1,2,\ldots ,6)$ with angle *θ*_*l*_.

4 $$\begin{array}{c}|H\rangle \,\mathop{\longrightarrow }\limits^{R({\theta }_{1})}\,\frac{1}{\sqrt{|{\alpha }_{00}{|}^{2}+|{\alpha }_{10}{|}^{2}}}({\alpha }_{00}|H\rangle +{\alpha }_{10}|V\rangle ),\\ |H\rangle \,\mathop{\longrightarrow }\limits^{R({\theta }_{2})}\,\frac{1}{\sqrt{|{\alpha }_{01}{|}^{2}+|{\alpha }_{11}{|}^{2}}}({\alpha }_{01}|H\rangle +{\alpha }_{11}|V\rangle )\\ |V\rangle \,\mathop{\longrightarrow }\limits^{R({\theta }_{3})}\,\frac{1}{\sqrt{|{\alpha }_{00}{|}^{2}+|{\alpha }_{10}{|}^{2}}}({\alpha }_{10}|H\rangle +{\alpha }_{00}|V\rangle ),\\ |V\rangle \,\mathop{\longrightarrow }\limits^{R({\theta }_{4})}\,\frac{1}{\sqrt{|{\alpha }_{01}{|}^{2}+|{\alpha }_{11}{|}^{2}}}({\alpha }_{11}|H\rangle +{\alpha }_{01}|V\rangle )\\ |H\rangle \,\mathop{\longrightarrow }\limits^{R({\theta }_{5})}\,\frac{1}{\sqrt{|{\alpha }_{00}{|}^{2}+|{\alpha }_{10}{|}^{2}}}({\alpha }_{10}|H\rangle +{\alpha }_{00}|V\rangle ),\\ |V\rangle \,\mathop{\longrightarrow }\limits^{R({\theta }_{6})}\,\frac{1}{\sqrt{|{\alpha }_{00}{|}^{2}+|{\alpha }_{10}{|}^{2}}}({\alpha }_{00}|H\rangle +{\alpha }_{10}|V\rangle ),\end{array}$$

where

5 $$\begin{array}{c}{\theta }_{1}=arccos\frac{{\alpha }_{00}}{\sqrt{|{\alpha }_{00}{|}^{2}+|{\alpha }_{10}{|}^{2}}},\\ {\theta }_{2}=arccos\frac{{\alpha }_{01}}{\sqrt{|{\alpha }_{01}{|}^{2}+|{\alpha }_{11}{|}^{2}}},\\ {\theta }_{3}=\pi -arcsin\frac{{\alpha }_{01}}{\sqrt{|{\alpha }_{10}{|}^{2}+|{\alpha }_{00}{|}^{2}}},\\ {\theta }_{4}=\pi -arcsin\frac{{\alpha }_{11}}{\sqrt{|{\alpha }_{11}{|}^{2}+|{\alpha }_{01}{|}^{2}}},\\ {\theta }_{5}=arccos\frac{{\alpha }_{10}}{\sqrt{|{\alpha }_{10}{|}^{2}+|{\alpha }_{00}{|}^{2}}},\\ {\theta }_{6}=\pi -arcsin\frac{{\alpha }_{00}}{\sqrt{|{\alpha }_{00}{|}^{2}+|{\alpha }_{10}{|}^{2}}}\mathrm{.}\end{array}$$

After photon A passes through wave plates *R*(*θ*_1_) and *R*(*θ*_*l*_) $(l=1,2,\ldots ,6)$ in spatial modes ${c}_{0},{c^{\prime} }_{0}$, ${d}_{0},{d^{\prime} }_{0}$, ${c}_{1},{c^{\prime} }_{1}$, ${d}_{1},{d^{\prime} }_{1}$, the state of photons A, B is changed from |Φ_1_〉_*AB*_ to |Φ_2_〉_*AB*_

6 $$\begin{array}{rcl}|{{\rm{\Phi }}}_{2}{\rangle }_{AB} & = & ({\alpha }_{00}|H{c}_{0}\rangle +{\alpha }_{01}|H{c}_{1}\rangle +{\alpha }_{10}|V{c}_{0}\rangle +{\alpha }_{11}|V{c}_{1}\rangle )|H{b}_{0}\rangle \\  &  & +\,({\alpha }_{01}|H{d}_{0}\rangle +{\alpha }_{10}|H{d}_{1}\rangle +{\alpha }_{11}|V{d}_{0}\rangle \\  &  & +\,{\alpha }_{00}|V{d}_{1}\rangle )|H{b}_{1}\rangle +({\alpha }_{10}|H{c^{\prime} }_{0}\rangle +{\alpha }_{11}|H{c^{\prime} }_{1}\rangle \\  &  & +\,{\alpha }_{00}|V{c^{\prime} }_{0}\rangle +{\alpha }_{01}|V{c^{\prime} }_{1}\rangle )|V{b}_{0}\rangle +({\alpha }_{11}|H{d^{\prime} }_{0}\rangle \\  &  & +\,{\alpha }_{00}|H{d^{\prime} }_{1}\rangle +{\alpha }_{01}|V{d^{\prime} }_{0}\rangle +{\alpha }_{10}|V{d^{\prime} }_{1}\rangle )|V{b}_{1}\rangle .\end{array}$$

To prepare the arbitrary two-qubit hybrid state remotely, the wavepackets from spatial modes *c*_0_, *d*_0_, ${c^{\prime} }_{0},{d^{\prime} }_{0}$ and *c*_1_, *d*_1_, ${c^{\prime} }_{1},{d^{\prime} }_{1}$ are put into BSs (i.e., *BS*_1_, *BS*_2_, *BS*_3_ and *BS*_4_)which are used to perform Hadamard operation on spatial-mode DOF.

7 $$\begin{array}{c}|{c}_{i}\rangle \to \frac{1}{2}(|{c}_{i}\rangle +|{c^{\prime} }_{i}\rangle +|{d}_{i}\rangle +|{d^{\prime} }_{i}\rangle ),\\ |{d}_{i}\rangle \to \frac{1}{2}(|{c}_{i}\rangle +|{c^{\prime} }_{i}\rangle -|{d}_{i}\rangle -|{d^{\prime} }_{i}\rangle )\\ |{c^{\prime} }_{i}\rangle \to \frac{1}{2}(|{c}_{i}\rangle -|{c^{\prime} }_{i}\rangle +|{d}_{i}\rangle -|{d^{\prime} }_{i}\rangle ),\\ |{d^{\prime} }_{i}\rangle \to \frac{1}{2}(|{c}_{i}\rangle -|{c^{\prime} }_{i}\rangle -|{d}_{i}\rangle +|{d^{\prime} }_{i}\rangle ),\end{array}$$

where i = 0, 1.

**Figure 2:**
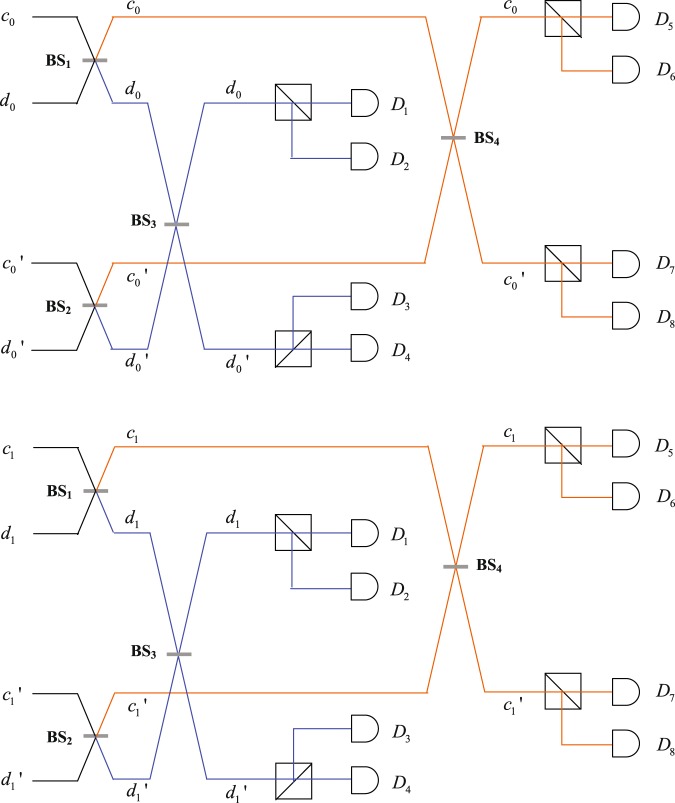
Quantum circuit for implementation of Hadamard operation via Beam splitters between spatial modes ${c}_{0},{c^{\prime} }_{0}$, ${d}_{0},{d^{\prime} }_{0}$ and ${c}_{1},{c^{\prime} }_{1}$, ${d}_{1},{d^{\prime} }_{1}$. ${c}_{0},{c^{\prime} }_{0}$, ${d}_{0},{d^{\prime} }_{0}$ and ${c}_{1},{c^{\prime} }_{1}$, ${d}_{1},{d^{\prime} }_{1}$ are spatial modes of photon A. BS represents a 50:50 beam splitter which implements a Hadamard operation on spatial-mode DOF.

The setup of implementation the Hadamard operation on spatial-mode DOF is shown in Fig. 2. The state of photons A, B evolves into |Φ_3_〉_*AB*_ after photon A in spatial modes *c*_0_, *d*_0_, ${c^{\prime} }_{0},{d^{\prime} }_{0}$ and *c*_1_, *d*_1_, ${c^{\prime} }_{1},{d^{\prime} }_{1}$ passes through BSs(without normalization).

8 $$\begin{array}{rcl}|{{\rm{\Phi }}}_{3}{\rangle }_{AB} & = & {\alpha }_{00}|H\rangle (|{c}_{0}\rangle +|{c^{\prime} }_{0}\rangle +|{d}_{0}\rangle +|{d^{\prime} }_{0}\rangle )|H{b}_{0}\rangle \\  &  & +\,{\alpha }_{01}|H\rangle (|{c}_{1}\rangle +|{c^{\prime} }_{1}\rangle +|{d}_{1}\rangle +|{d^{\prime} }_{1}\rangle )|H{b}_{0}\rangle \\  &  & +\,{\alpha }_{10}|V\rangle (|{c}_{0}\rangle +|{c^{\prime} }_{0}\rangle +|{d}_{0}\rangle +|{d^{\prime} }_{0}\rangle )|H{b}_{0}\rangle \\  &  & +\,{\alpha }_{11}|V\rangle (|{c}_{1}\rangle +|{c^{\prime} }_{1}\rangle +|{d}_{1}\rangle +|{d^{\prime} }_{1}\rangle )|H{b}_{0}\rangle \\  &  & +\,{\alpha }_{01}|H\rangle (|{c}_{0}\rangle +|{c^{\prime} }_{0}\rangle -|{d}_{0}\rangle -|{d^{\prime} }_{0}\rangle )|H{b}_{1}\rangle \\  &  & +\,{\alpha }_{10}|H\rangle (|{c}_{1}\rangle +|{c^{\prime} }_{1}\rangle -|{d}_{1}\rangle -|{d^{\prime} }_{1}\rangle )|H{b}_{1}\rangle \\  &  & +\,{\alpha }_{11}|V\rangle (|{c}_{0}\rangle +|{c^{\prime} }_{0}\rangle -|{d}_{0}\rangle -|{d^{\prime} }_{0}\rangle )|H{b}_{1}\rangle \\  &  & +\,{\alpha }_{00}|V\rangle (|{c}_{1}\rangle +|{c^{\prime} }_{1}\rangle -|{d}_{1}\rangle -|{d^{\prime} }_{1}\rangle )|H{b}_{1}\rangle \\  &  & +\,{\alpha }_{10}|H\rangle (|{c}_{0}\rangle -|{c^{\prime} }_{0}\rangle +|{d}_{0}\rangle -|{d^{\prime} }_{0}\rangle )|V{b}_{0}\rangle \\  &  & +\,{\alpha }_{11}|H\rangle (|{c}_{1}\rangle -|{c^{\prime} }_{1}\rangle +|{d}_{1}\rangle -|{d^{\prime} }_{1}\rangle )|V{b}_{0}\rangle \\  &  & +\,{\alpha }_{00}|V\rangle (|{c}_{0}\rangle -|{c^{\prime} }_{0}\rangle +|{d}_{0}\rangle -|{d^{\prime} }_{0}\rangle )|V{b}_{0}\rangle \\  &  & +\,{\alpha }_{01}|V\rangle (|{c}_{1}\rangle -|{c^{\prime} }_{1}\rangle +|{d}_{1}\rangle -|{d^{\prime} }_{1}\rangle )|V{b}_{0}\rangle \\  &  & +\,{\alpha }_{11}|H\rangle (|{c}_{0}\rangle -|{c^{\prime} }_{0}\rangle -|{d}_{0}\rangle +|{d^{\prime} }_{0}\rangle )|V{b}_{1}\rangle \\  &  & +\,{\alpha }_{00}|H\rangle (|{c}_{1}\rangle -|{c^{\prime} }_{1}\rangle -|{d}_{1}\rangle +|{d^{\prime} }_{1}\rangle )|V{b}_{1}\rangle \\  &  & +\,{\alpha }_{01}|V\rangle (|{c}_{0}\rangle -|{c^{\prime} }_{0}\rangle -|{d}_{0}\rangle +|{d^{\prime} }_{0}\rangle )|V{b}_{1}\rangle \\  &  & +\,{\alpha }_{10}|V\rangle (|{c}_{1}\rangle -|{c^{\prime} }_{1}\rangle -|{d}_{1}\rangle +|{d^{\prime} }_{1}\rangle )|V{b}_{1}\rangle \end{array}$$

Alice performs single particle measurement on photon A, and the original two-qubit state can be remotely prepared onto Bob’s hyperentangled photon B if Bob applies the appropriate recovery operations. In detail, the relation between Alice’s single-particle measurement result, the state of photon B in the hand of Bob after the single-particle measurement done by Alice and the unitary operation with which Bob can prepare the original state |Ψ〉 is shown in Table 1. Here I is the identity matrix, ${\sigma }_{x}^{p}$, ${\sigma }_{z}^{p}$ are the pauli matrices of polarization DOF and ${\sigma }_{x}^{s}$, ${\sigma }_{z}^{s}$ are the pauli matrices of spatial-mode DOF.

9 $$\begin{array}{c}{\sigma }_{x}^{p}=|H\rangle \langle V|+|V\rangle \langle H|,\\ {\sigma }_{z}^{p}=|H\rangle \langle H|-|V\rangle \langle V|,\\ {\sigma }_{x}^{s}=|{b}_{0}\rangle \langle {b}_{1}|+|{b}_{1}\rangle \langle {b}_{0}|,\\ {\sigma }_{z}^{s}=|{b}_{0}\rangle \langle {b}_{0}|-|{b}_{1}\rangle \langle {b}_{1}|.\end{array}$$

**Table 1:** The relation between Alice’s single-particle measurement result, the state of photon B in the hand of Bob after the single-particle measurement done by Alice and the unitary operation with which the original state |Ψ〉 can be remote prepared.

Alice’s measurement result	The state of photon B	Unitary operation
|*Hc*_0_〉	*α*00|*Hb*0〉 + *α*01|*Hb*1〉 + *α*10|*Vb*0〉 + *α*11|*Vb*1〉	I
$|H{c^{\prime} }_{0}\rangle$	*α*_00_|*Hb*_0_〉 + *α*_01_|*Hb*_1_〉 − *α*_10_|*Vb*_0_〉 − *α*_11_|*Vb*_1_〉	${\sigma }_{z}^{p}$
|*Hd*_0_〉	*α*_00_|*Hb*_0_〉 − *α*_01_|*Hb*_1_〉 + *α*_10_|*Vb*_0_〉 − *α*_11_|*Vb*_1_〉	$${\sigma }_{z}^{s}$$
$|H{d^{\prime} }_{0}\rangle$	*α*_00_|*Hb*_0_〉 − *α*_01_|*Hb*_1_〉 − *α*_10_|*Vb*_0_〉 + *α*_11_|*Vb*_1_〉	${\sigma }_{z}^{s}{\sigma }_{z}^{p}$
|*Hc*_1_〉	*α*_01_|*Hb*_0_〉 + *α*_10_|*Hb*_1_〉 + *α*_11_|*Vb*_0_〉 + *α*_00_|*Vb*_1_〉	$${({\sigma }_{x}^{p})}_{{b}_{0}}{\sigma }_{x}^{s}$$
$|H{c^{\prime} }_{1}\rangle$	*α*_01_|*Hb*_0_〉 + *α*_10_|*Hb*_1_〉 − *α*_11_|*Vb*_0_〉 − *α*_00_|*Vb*_1_〉	${({\sigma }_{x}^{p})}_{{b}_{0}}{\sigma }_{x}^{s}{\sigma }_{z}^{p}$
|*Hd*_1_〉	*α*_01_|*Hb*_0_〉 − *α*_10_|*Hb*_1_〉 + *α*_11_|*Vb*_0_〉 − *α*_00_|*Vb*_1_〉	$${({\sigma }_{x}^{p})}_{{b}_{0}}{\sigma }_{x}^{s}{\sigma }_{z}^{s}$$
$|H{d^{\prime} }_{1}\rangle$	*α*_01_|*Hb*_0_〉 − *α*_10_|*Hb*_1_〉 − *α*_11_|*Vb*_0_〉 + *α*_00_|*Vb*_1_〉	${({\sigma }_{x}^{p})}_{{b}_{0}}{\sigma }_{x}^{s}{\sigma }_{z}^{s}{\sigma }_{z}^{p}$
|*Vc*_0_〉	*α*_10_|*Hb*_0_〉 + *α*_11_|*Hb*_1_〉 + *α*_00_|*Vb*_0_〉 + *α*_01_|*Vb*_1_〉	$${\sigma }_{x}^{p}$$
$|V{c^{\prime} }_{0}\rangle$	*α*_10_|*Hb*_0_〉 + *α*_11_|*Hb*_1_〉 − *α*_00_|*Vb*_0_〉 − *α*_01_|*Vb*_1_〉	${\sigma }_{x}^{p}{\sigma }_{z}^{p}$
|*Vd*_0_〉	*α*_10_|*Hb*_0_〉 − *α*_11_|*Hb*_1_〉 + *α*_00_|*Vb*_0_〉 − *α*_01_|*Vb*_1_〉	$${\sigma }_{x}^{p}{\sigma }_{z}^{s}$$
$|V{d^{\prime} }_{0}\rangle$	*α*_10_|*Hb*_0_〉 − *α*_11_|*Hb*_1_〉 − *α*_00_|*Vb*_0_〉 + *α*_01_|*Vb*_1_〉	${\sigma }_{x}^{p}{\sigma }_{z}^{s}{\sigma }_{z}^{p}$
|*Vc*_1_〉	*α*_11_|*Hb*_0_〉 + *α*_00_|*Hb*_1_〉 + *α*_01_|*Vb*_0_〉 + *α*_10_|*Vb*_1_〉	$${({\sigma }_{x}^{p})}_{{b}_{1}}{\sigma }_{x}^{s}$$
$|V{c^{\prime} }_{1}\rangle$	*α*_11_|*Hb*_0_〉 + *α*_00_|*Hb*_1_〉 − *α*_01_|*Vb*_0_〉 − *α*_10_|*Vb*_1_〉	${({\sigma }_{x}^{p})}_{{b}_{1}}{\sigma }_{x}^{s}{\sigma }_{z}^{p}$
|*Vd*_1_〉	*α*_11_|*Hb*_0_〉 − *α*_00_|*Hb*_1_〉 + *α*_01_|*Vb*_0_〉 − *α*_10_|*Vb*_1_〉	$${({\sigma }_{x}^{p})}_{{b}_{1}}{\sigma }_{x}^{s}{\sigma }_{z}^{s}$$
$|V{d^{\prime} }_{1}\rangle$	*α*_11_|*Hb*_0_〉 − *α*_00_|*Hb*_1_〉 − *α*_01_|*Vb*_0_〉 + *α*_10_|*Vb*_1_〉	${({\sigma }_{x}^{p})}_{{b}_{1}}{\sigma }_{x}^{s}{\sigma }_{z}^{s}{\sigma }_{z}^{p}$

${({\sigma }_{x}^{p})}_{{b}_{0}}$ represents implements a polarization bit-flip operation ${\sigma }_{x}^{p}$ in spatial mode *b*_0_ and ${({\sigma }_{x}^{p})}_{{b}_{1}}$ represents implements a polarization bit-flip operation ${\sigma }_{x}^{p}$ in spatial mode *b*_1_.

As discussed in ref.^64^, the original two-qubit hybrid state |Ψ〉 can be remotely prepared onto the receiver’s hyperentangled photon by letting Bob know the corresponding unitary operation via 4 cbits. That is, the original two-qubit hybrid state can be remote prepared with a cost of 2 ebits and 4 cbits.

#### Two-qubit hybrid state remote preparation via partially hyperentangled Bell state

Now, let us discuss the recursive remote preparation protocol for two-qubit hybrid state via partially hyperentangled Bell state with linear optics. Similarly, in remote preparation of two-qubit hybrid state via partially hyperentangled Bell state, the coefficients of two-qubit hybrid state |Ψ〉 = *α*_00_|*Ha*_0_〉 + *α*_01_|*Ha*_1_〉 + *α*_10_|*Va*_0_〉 + *α*_11_|*Va*_1_〉 is completely known by the sender Alice but unknown by the receiver Bob. Alice wants to help the remote receiver prepare the hybrid state. To prepare the original two-qubit hybrid state remotely, Alice first pre-adjusts the partially hyperentangled state to a target quantum channel in accordance with his knowledge of the original two-qubit state |Ψ〉 via linear-optical elements, and then performs single-particle measurement on his hyperentangled photon. The original two-qubit hybrid state can be remotely prepared onto Bob’s hyperentangled photon B by applying appropriate recovery operations.

Suppose the quantum channel shared by the sender Alice and the receiver Bob is a two-photon four-qubit partially hyperentangled state^47^

10 $$\begin{array}{rcl}|{\rm{\Phi }}^{\prime} \rangle  & = & ({\beta }_{0}|HH\rangle +{\beta }_{1}|VV\rangle )\otimes ({\gamma }_{0}|{a}_{0}{b}_{0}\rangle +{\gamma }_{1}|{a}_{1}{b}_{1}\rangle )\\  & = & {\beta }_{0}{\gamma }_{0}|H{a}_{0}\rangle |H{b}_{0}\rangle +{\beta }_{0}{\gamma }_{1}|H{a}_{1}\rangle |H{b}_{1}\rangle \\  &  & +\,{\beta }_{1}{\gamma }_{0}|V{a}_{0}\rangle |V{b}_{0}\rangle +{\beta }_{1}{\gamma }_{1}|V{a}_{1}\rangle |V{b}_{1}\rangle \end{array}$$

where |*β*_0_|^2^ + |*β*_0_|^2^ = 1, |*γ*_0_|^2^ + *γ*_0_|^2^ = 1.

**Figure 3:**
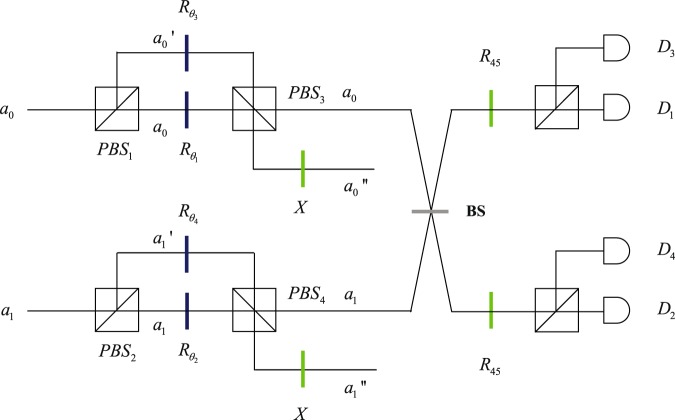
Schematic diagram of the setup for two-qubit hybrid state remote preparation via partially hyperentangled Bell state. *a*_0_, *a*_1_ are two spatial modes of photon A. The wave plates ${R}_{{\theta }_{1}},{R}_{{\theta }_{2}},{R}_{{\theta }_{3}},{R}_{{\theta }_{4}}$ rotate the photon horizontal and vertical polarizations with angles *θ*_1_, *θ*_2_, *θ*_3_, *θ*_4_. X represents a half-wave plate which can implement a polarization bit-flip operation ${\sigma }_{x}^{p}=|H\rangle \langle V|+|V\rangle \langle H|$. BS represents a 50:50 beam splitter which implements a Hadamard operation on spatial-mode DOF. The wave plate *R*_45_ rotates the photon horizontal and vertical polarizations with angles *θ*_1_, which implements a Hadamard operation on polarization DOF.

The setup of two-qubit hybrid state remote preparation with hyperentangled Bell state via linear optics is shown in Fig. 3. Alice rotates the polarization state of photon A via wave plates ${R}_{{\theta }_{1}},{R}_{{\theta }_{2}},{R}_{{\theta }_{3}},{R}_{{\theta }_{4}}$ in spatial modes *a*_0_, ${a^{\prime} }_{0}$, *a*_1_, ${a^{\prime} }_{1}$ with angles *θ*_1_, *θ*_2_, *θ*_3_, *θ*_4_.

11 $$\begin{array}{c}|H{a}_{0}\rangle \,\mathop{\longrightarrow }\limits^{R({\theta }_{1})}\,({R}_{1}|H\rangle +\sqrt{1-{R}_{1}^{2}}|V\rangle )|{a}_{0}\rangle ,\\ |H{a}_{1}\rangle \,\mathop{\longrightarrow }\limits^{R({\theta }_{2})}\,({R}_{2}|H\rangle +\sqrt{1-{R}_{2}^{2}}|V\rangle )|{a}_{1}\rangle \\ |V{a^{\prime} }_{0}\rangle \,\mathop{\longrightarrow }\limits^{R({\theta }_{3})}\,({R}_{3}|V\rangle +\sqrt{1-{R}_{3}^{2}}|H\rangle )|{a^{\prime} }_{0}\rangle ,\\ |V{a^{\prime} }_{1}\rangle \,\mathop{\longrightarrow }\limits^{R({\theta }_{4})}\,({R}_{4}|V\rangle +\sqrt{1-{R}_{4}^{2}}|H\rangle )|{a^{\prime} }_{1}\rangle \end{array}$$

where

12 $$\begin{array}{c}{R}_{1}=\frac{\frac{{\alpha }_{00}}{{\beta }_{0}{\gamma }_{0}}}{\sqrt{{(\frac{{\alpha }_{00}}{{\beta }_{0}{\gamma }_{0}})}^{2}+{(\frac{{\alpha }_{01}}{{\beta }_{0}{\gamma }_{1}})}^{2}+{\frac{{\alpha }_{10}}{{\beta }_{1}{\gamma }_{0}})}^{2}+{\frac{{\alpha }_{11}}{{\beta }_{1}{\gamma }_{1}})}^{2}}},\\ {R}_{2}=\frac{\frac{{\alpha }_{01}}{{\beta }_{0}{\gamma }_{1}}}{\sqrt{{(\frac{{\alpha }_{00}}{{\beta }_{0}{\gamma }_{0}})}^{2}+{(\frac{{\alpha }_{01}}{{\beta }_{0}{\gamma }_{1}})}^{2}+{\frac{{\alpha }_{10}}{{\beta }_{1}{\gamma }_{0}})}^{2}+{\frac{{\alpha }_{11}}{{\beta }_{1}{\gamma }_{1}})}^{2}}}\\ {R}_{3}=\frac{\frac{{\alpha }_{10}}{{\beta }_{1}{\gamma }_{0}}}{\sqrt{{(\frac{{\alpha }_{00}}{{\beta }_{0}{\gamma }_{0}})}^{2}+{(\frac{{\alpha }_{01}}{{\beta }_{0}{\gamma }_{1}})}^{2}+{\frac{{\alpha }_{10}}{{\beta }_{1}{\gamma }_{0}})}^{2}+{\frac{{\alpha }_{11}}{{\beta }_{1}{\gamma }_{1}})}^{2}}},\\ {R}_{4}=\frac{\frac{{\alpha }_{11}}{{\beta }_{1}{\gamma }_{1}}}{\sqrt{{(\frac{{\alpha }_{00}}{{\beta }_{0}{\gamma }_{0}})}^{2}+{(\frac{{\alpha }_{01}}{{\beta }_{0}{\gamma }_{1}})}^{2}+{\frac{{\alpha }_{10}}{{\beta }_{1}{\gamma }_{0}})}^{2}+{\frac{{\alpha }_{11}}{{\beta }_{1}{\gamma }_{1}})}^{2}}}\end{array}$$

and

13 $$\begin{array}{c}{\theta }_{1}=arccos{R}_{1},\\ {\theta }_{2}=arccos{R}_{2},\\ {\theta }_{3}=arcsin{R}_{3}-\frac{\pi }{2},\\ {\theta }_{4}=arcsin{R}_{4}-\frac{\pi }{2}\mathrm{.}\end{array}$$

One can get the two-photon system in the state $|{{\rm{\Phi }}^{\prime} }_{1}\rangle$ after photon A passes through wave plates ${R}_{{\theta }_{1}},{R}_{{\theta }_{2}},{R}_{{\theta }_{3}},{R}_{{\theta }_{4}}$ in spatial modes *a*_0_, ${a^{\prime} }_{0}$, *a*_1_, ${a^{\prime} }_{1}$.

14 $$\begin{array}{rcl}|{{\rm{\Phi }}^{\prime} }_{1}\rangle  & = & {\beta }_{0}{\gamma }_{0}({R}_{1}|H\rangle +\sqrt{1-{R}_{1}^{2}}|V\rangle )|{a}_{0}\rangle |H{b}_{0}\rangle \\  &  & +\,{\beta }_{0}{\gamma }_{1}({R}_{2}|H\rangle +\sqrt{1-{R}_{2}^{2}}|V\rangle )|{a}_{1}\rangle |H{b}_{1}\rangle \\  &  & +\,{\beta }_{1}{\gamma }_{0}({R}_{3}|V\rangle +\sqrt{1-{R}_{3}^{2}}|H\rangle )|{a^{\prime} }_{0}\rangle |V{b}_{0}\rangle \\  &  & +\,{\beta }_{1}{\gamma }_{1}({R}_{4}|V\rangle +\sqrt{1-{R}_{4}^{2}}|H\rangle )|{a^{\prime} }_{1}\rangle |V{b}_{1}\rangle .\end{array}$$

After photon A passes through *PBS*_3_, *PBS*_4_, the state of composite system is transformed from |Φ′〉_1_ to |Φ′〉_2_ if photon A is emitted from spatial modes *a*_0_, *a*_1_.

15 $$\begin{array}{rcl}|{{\rm{\Phi }}^{\prime} }_{2}\rangle  & = & {\alpha }_{00}|H{a}_{0}\rangle |H{b}_{0}\rangle +{\alpha }_{01}|H{a}_{1}\rangle |H{b}_{1}\rangle \\  &  & +\,{\alpha }_{10}|V{a}_{0}\rangle |V{b}_{0}\rangle +{\alpha }_{11}|V{a}_{1}\rangle |V{b}_{1}\rangle .\end{array}$$

The transformation of quantum channel is succeeds if photon A is emitted from spatial modes *a*_0_, *a*_1_.

To remote prepare the original state, Alice performs single-qubit X-basis measurement on polarization and spatial-mode DOFs. To perform the X-basis measurement on polarization and spatial-mode DOFs, Alice first implements the Hadamard operations on polarization and spatial-mode DOFs, then performs single-qubit measurements. That is, the state of composite system is changed from $|{{\rm{\Phi }}^{\prime} }_{2}\rangle$ to $|{{\rm{\Phi }}^{\prime} }_{3}\rangle$ after photon A passes through BSs and wave plates *R*_45_.(without normalization)

16 $$\begin{array}{rcl}|{{\rm{\Phi }}^{\prime} }_{3}\rangle  & = & |H{a}_{0}\rangle ({\alpha }_{00}|H{b}_{0}\rangle +{\alpha }_{01}|H{b}_{1}\rangle +{\alpha }_{10}|V{b}_{0}\rangle +{\alpha }_{11}|V{b}_{1}\rangle )\\  &  & +\,|H{a}_{1}\rangle ({\alpha }_{00}|H{b}_{0}\rangle -{\alpha }_{01}|H{b}_{1}\rangle +{\alpha }_{10}|V{b}_{0}\rangle -{\alpha }_{11}|V{b}_{1}\rangle )\\  &  & +\,|V{a}_{0}\rangle ({\alpha }_{00}|H{b}_{0}\rangle +{\alpha }_{01}|H{b}_{1}\rangle -{\alpha }_{10}|V{b}_{0}\rangle -{\alpha }_{11}|V{b}_{1}\rangle )\\  &  & +\,|V{a}_{1}\rangle ({\alpha }_{00}|H{b}_{0}\rangle -{\alpha }_{01}|H{b}_{1}\rangle -{\alpha }_{10}|V{b}_{0}\rangle +{\alpha }_{11}|V{b}_{1}\rangle ).\end{array}$$

The state of photon B collapses to *α*_00_|*Hb*_0_〉 + *α*_01_|*Hb*_1_〉 + *α*_10_|*Vb*_0_〉 + *α*_11_|*Vb*_1_〉, *α*_00_|*Hb*_0_〉 − *α*_01_|*Hb*_1_〉 + *α*_10_|*Vb*_0_〉 − *α*_11_|*Vb*_1_〉, *α*_00_|*Hb*_0_〉 + *α*_01_|*Hb*_1_〉 − *α*_10_|*Vb*_0_〉 − *α*_11_|*Vb*_1_〉 or *α*_00_|*Hb*_0_〉 − *α*_01_|*Hb*_1_〉 − *α*_10_|*Vb*_0_〉 + *α*_11_|*Vb*_1_〉 if Alice’s single-qubit measurement result is |*Ha*_0_〉, |*Ha*_1_〉, |*Va*_0_〉 or |*Va*_1_〉, respectively. The state |Φ〉 can be remotely prepared onto Bob’s hyperentangled photon B by applying appropriate recovery operation *I*, ${\sigma }_{z}^{s}$, ${\sigma }_{z}^{p}$ or ${\sigma }_{z}^{s}{\sigma }_{z}^{p}$.

If the quantum channel transformation fails in this round. That is, photon A is emitted from spatial modes ${a^{\prime\prime} }_{0}$, ${a^{\prime\prime} }_{1}$. The state of composite system composed of photons A, B is transformed from |Φ′〉_1_ to |*ϕ*〉 if photon A is emitted from spatial modes ${a^{\prime\prime} }_{0}$, ${a^{\prime\prime} }_{1}$.

17 $$\begin{array}{rcl}|\varphi \rangle  & = & {\beta }_{0}{\gamma }_{0}\sqrt{1-{R}_{1}^{2}}|V{a^{\prime\prime} }_{0}\rangle |H{b}_{0}\rangle +{\beta }_{0}{\gamma }_{1}\sqrt{1-{R}_{2}^{2}}|V{a^{\prime\prime} }_{1}\rangle |H{b}_{1}\rangle \\  &  & +\,{\beta }_{1}{\gamma }_{0}\sqrt{1-{R}_{3}^{2}}|H{a^{\prime\prime} }_{0}\rangle |V{b}_{0}\rangle \\  &  & +\,{\beta }_{1}{\gamma }_{1}\sqrt{1-{R}_{4}^{2}}|H{a^{\prime\prime} }_{1}\rangle |V{b}_{1}\rangle .\end{array}$$

To transform the quantum channel recursively, Alice performs bit-flip operation ${\sigma }_{x}^{p}$ on polarization DOF via the half-wave plate X. The state of composite system is changed from |*ϕ*〉 to |*ϕ*_1_〉 after photon A passes through half-wave plate X.

18 $$\begin{array}{rcl}|{\varphi }_{1}\rangle  & = & {\beta }_{0}{\gamma }_{0}\sqrt{1-{R}_{1}^{2}}|H{a^{\prime\prime} }_{0}\rangle |H{b}_{0}\rangle +{\beta }_{0}{\gamma }_{1}\sqrt{1-{R}_{2}^{2}}|H{a^{\prime\prime} }_{1}\rangle |H{b}_{1}\rangle \\  &  & +\,{\beta }_{1}{\gamma }_{0}\sqrt{1-{R}_{3}^{2}}|V{a^{\prime\prime} }_{0}\rangle |V{b}_{0}\rangle \\  &  & +\,{\beta }_{1}{\gamma }_{1}\sqrt{1-{R}_{4}^{2}}|V{a^{\prime\prime} }_{1}\rangle |V{b}_{1}\rangle .\end{array}$$

One can see the hyperentangled state |*ϕ*_1_〉 is still in the manner of partially hyperentangled Bell state with different coefficients^68^. The sender Alice can transform the quantum channel recursively until the transformation succeeds. In this sense, Alice can remotely prepare the hybrid state |Ψ〉 onto the receiver’s quantum system via partially hyperentangled Bell state |Φ′〉 and and linear optics by transforming the partially hyperentangled channel in a recursive manner. As the sender only needs linear-optical elements to realize parallel remote state preparation and the partially hyperentangled quantum channel can be transformed recursively, this RSP scheme is more convenient in application than others^69^.

## Discussion

In the present remote state preparation protocol, we use hyperentangled Bell state which entangled in polarization and spatial-mode DOFs as the quantum channel for remote preparation of an arbitrary two-qubit hybrid state. By using this hyperentangled state, one can perform quantum teleportation^58^, quantum entanglement swapping^70^ and parallel remote state preparation^69^. However, if one uses the partially hyperentangled state to prepare quantum state remotely, the fidelity of prepared state will be reduced since the quantum channel noise. In the current protocol, this problem does not exist because the partially hyperentangled quantum channel is transformed recursively via linear optics until the transformation succeeds.

In conclusion, we have proposed a remote preparation protocol for two-qubit hybrid state with hyperentangled Bell state, resorting to linear-optical elements. In our protocol, the hybrid state to be prepared can be remotely reconstruct via linear-optical elements with a cost of 4 cbits and 2 ebits, which is very different from previous two-qubit state remote preparation protocol^64^. With the UBS and the wave plate on spatial-mode DOF and polarization DOF, we show that the target hyperentangled quantum channel for two-qubit hybrid state remote preparation can be obtained, where the spatial-mode state and polarization state of hyperentangled photon is rotated according to the sender’s information of prepared state. Moreover, we show the remote state preparation protocol for two-qubit hybrid state via partially hyperentangled Bell state, resorting to linear-optical elements only. As it requires only linear-optical elements and has a less classical communication cost, our protocol is practical and efficient for preparing quantum state remotely for long-distance quantum communication.

## Methods

### UBS

The set up for our UBS is shown in Fig. 1(a). If spatial-mode state of input photon is |*a*_0_〉, the spatial-mode state of photon is transformed into corresponding state after photon A passes through the beam splitters and the wave plate *ω*.

19 $$\begin{array}{lll}|{a}_{0}\rangle  & \mathop{\longrightarrow }\limits^{B{S}_{1},\omega } & {e}^{i\omega }|{a}_{1}\rangle +i|{a}_{0}\rangle \\  & \mathop{\longrightarrow }\limits^{B{S}_{2}} & {e}^{i\omega }(|{a}_{0}\rangle +i|{a}_{1}\rangle )+i(|{a}_{1}\rangle +i|{a}_{0}\rangle )\\  & = & sin\frac{\omega }{2}|{a}_{0}\rangle +cos\frac{\omega }{2}|{a}_{1}\rangle \end{array}$$

If spatial-mode state of input photon is |*a*_1_〉, the spatial-mode state of photon is rotated after photon A passes through the beam splitters and the wave plate *ω*.

20 $$\begin{array}{lll}|{a}_{1}\rangle  & \mathop{\longrightarrow }\limits^{B{S}_{1},\omega } & i{e}^{i\omega }|{a}_{1}\rangle +|{a}_{0}\rangle \\  & \mathop{\longrightarrow }\limits^{B{S}_{2}} & i{e}^{i\omega }(|{a}_{0}\rangle +i|{a}_{1}\rangle )+(|{a}_{1}\rangle +i|{a}_{0}\rangle )\\  & = & cos\frac{\omega }{2}|{a}_{0}\rangle -sin\frac{\omega }{2}|{a}_{1}\rangle \end{array}$$

This is just the result of UBS.
